# I-PASS-Based Handoff Pilot in a High-Volume Urban Hospital: Benefits and Barriers for Hospitalists

**DOI:** 10.7759/cureus.80040

**Published:** 2025-03-04

**Authors:** Malavika Kapuria, Jasmah Hanna, Mary Ann Kirkconnell Hall, Simone Afriyie, Sarah Wondmeneh, Adekunle Obisesan, Reena Hemrajani, Amy Miller

**Affiliations:** 1 Division of Hospital Medicine, Emory University School of Medicine, Atlanta, USA; 2 Department of Medicine, Emory University School of Medicine, Atlanta, USA; 3 School of Medicine, Emory University, Atlanta, USA; 4 Hospital Medicine, Atlanta Veterans Affairs Medical Center, Atlanta, USA

**Keywords:** electronic health records, hospital-based medicine, hospitalists, patient handoffs, qualitative studies, standardized handoff, transitions in care

## Abstract

Background

The I-PASS bundle is a standardized care handoff associated with improved outcomes that is widely used in residency training. Sustainable use of I-PASS bundle components among attending hospital medicine providers merits further study. After we implemented a handoffs pilot process including written handoffs and Epic chat message (surrogate for verbal handoff) based on the I-PASS protocol, providers reported high levels of satisfaction. However, the pilot tool was utilized infrequently, and use ceased after the pilot.

Methodology

We utilized qualitative methods to assess attending physicians’ and Advanced Practice Providers’ perceptions to understand discrepancies between perceived utility and actual use. From February to March 2022, we interviewed 13 attending hospitalist physicians (n = 11) and Advanced Practice Providers (n = 2) individually or in homogenous focus groups; we transcribed recordings for qualitative coding (interrater agreement was κ = 0.82). We coded and analyzed the textual data via the specific concerns participants shared in the interviews and focus groups.

Results

Participants felt that while the I-PASS-based tool was suboptimal, the pilot raised overall awareness and use of handoff processes. They recommended that feasible handoff processes provide necessary information quickly for cross-cover providers, but do not require similar details for stable patients. Most participants reported that existing electronic medical record chat functions along with notes were adequate and more efficient. They also recommended standardizing format and processes; obtaining buy-in from all patient care providers; education on efficient use of tools; and formal, explicit expectations that providers complete handoffs.

Conclusions

Handoffs are an important component of patient safety measures. While they perceived the I-PASS-based tool to be suboptimal in several aspects, participants felt that the handoff quality improvement pilot raised overall awareness and use of handoff processes, which they felt was important for patient safety. Implementation of I-PASS bundle components may require contextual adaptation.

## Introduction

National guidelines recommend hospitals implement standardized approaches to handoffs [1-3]. One such set of interventions, the I-PASS bundle, has been widely adopted for shift handoffs. Its initial implementation among residents was associated with a 23% reduction in medical errors and a 30% reduction in preventable adverse events [2]. Recent quantitative research has indicated that standardized approaches such as the I-PASS bundle can lead to sustained improvements in patient safety and provider communication in a diverse array of settings [4-6]. Improvements in perceptions of patient care and comfort at the end of life [7] and satisfaction of providers and families after implementation of the I-PASS bundle [8] have also been reported.

I-PASS is an evidence-based handoff program designed to improve communication at times of shift handoff between healthcare providers. I-PASS is a mnemonic including illness severity, patient summary, action list, situational awareness, and synthesis by receiver. When utilized with a bundle of implementation in residency programs, this has been validated to reduce medical errors [2]. While the benefits and successful I-PASS implementation for resident physicians are clear [9], the sustainability of the I-PASS tool with more experienced physicians and in other settings merits consideration. Price et al. noted the need for research on approaches that balance the needs of individual facilities (e.g., teaching hospitals) with the most relevant components of the bundle [10].

The hospital medicine group at our facility has only existed since 2017, and at the time of the pilot described in this paper, we did not have an existing standard process for handoffs. Our hospital medicine group piloted a formal written handoffs process in our Epic (Epic, Verona, WI) electronic medical record (EMR) and an Epic chat message based on the I-PASS mnemonic with limited success. An Epic chat message is a live text message between one provider and allows real-time communication, with an option to attach information directly to a patient chart for reference. This message remains visible for several days. Implementation of all components of the full bundle at our site was not feasible, so we chose to prioritize the I-PASS mnemonic and EMR integration.

We conducted a five-point Likert scale survey of 30 hospitalist physicians and Advanced Practice Providers on the hospital medicine service at our hospital. The survey response rate was 70%; among those, 79% reported satisfaction with shift handoffs, but the pilot written tool was utilized just 45% of the time during the evaluated period. Upon completion of the formal quality improvement (QI) project, use of the written tool ceased, despite integration into the EMR and anecdotal continued use of the Epic chat function. This study aimed to use qualitative methods to evaluate hospitalists’ attitudes toward the handoff QI project, to understand barriers to adherence to the I-PASS-based tool in a large, high-volume urban hospital, and to evaluate hospitalists’ attitudes regarding best practices for shift handoffs.

An abstract with preliminary findings from this study was presented at the Emory University Division of Hospital Medicine Research Day, Atlanta, GA on August 11, 2022; the Southern Hospital Medicine annual meeting, New Orleans, LA, on October 14, 2022; the Emory University Department of Medicine Research Day, Atlanta, GA on October 27, 2022; and at the Society of Hospital Medicine Converge Annual Meeting, Austin, TX on March 28, 2023.

## Materials and methods

In this study, 13 participants (11 physicians and two Advanced Practice Providers) covering both day and night shifts participated in four interviews and two homogeneous focus groups in February and March 2022 (Table 1) using structured guides with questions derived from our observation of the results of the short survey of users following the pilot (Table 2). At the time of the study, our group had 33 physicians and four Advanced Practice Providers, so the participation rate was 35% overall, and 33% and 50% for physicians and Advanced Practice Providers, respectively.

**Table 1:** Focus group and interview participant demographics.

Data collection setting	Participants (N)	Participant occupations
Focus Group #1	4	Advanced Practice Provider/Nocturnist (n = 1); Physician/Daytime (n = 3)
Focus Group #2	5	Physician/Daytime (n = 5)
Interview #1	1	Physician/Daytime
Interview #2	1	Physician/Daytime
Interview #3	1	Advanced Practice Provider/Nocturnist
Interview #4	1	Physician/Daytime

**Table 2:** Focus group and interview question guide.

Question	Text
1	Should there be a shift handoff process?
1a	Why or why not?
2	If you were to design a shift handoff process, what would it look like ideally?
3	What is your experience with shift handoffs prior to the implementation of this quality improvement project?
4	What did you think of the shift handoff project implemented for the Grady hospitalist group?
4a	What worked well? What did not work well (would you change)?
5	We saw an improvement in satisfaction with shift handoffs during the duration of the project – why do you think this was?
6	We noticed a lower uptake of written handoff – what were reasons you had to use this or not use this?

We obtained leadership buy-in and requested a convenience sample of volunteers at staff meetings and via informal communications. The focus groups were announced at staff meetings followed by recruitment email invitations to providers who had participated in the QI project. We intentionally recruited nocturnists/cross-cover providers. We grouped participants into focus groups based on their self-reported general opinion regarding shift handoff (i.e., helpful vs. unhelpful) on the focus group Registration Form to allow for homogenous discussion. Participants completed interviews if they were unable to attend scheduled focus group sessions. We offered $10 USD gift cards to incentivize participation. No interview participants joined the focus groups or vice versa. All interviews were <30 minutes; the focus groups were 40 and 50 minutes long.

Focus groups and interviews were conducted remotely with participants and interviewers using Zoom software (Zoom Video Communications, Inc., San Jose, CA). Focus groups were moderated by a resident physician (author SW) who did not work in the facility where participants worked. The interviews were conducted by two authors (JH and MAKH) who were not involved with the pilot, but who were trained qualitative interviewers and analysts (an Associate Director of Research Projects with an MS degree and a Senior Medical Writer with an MPH). While some participants were familiar with the interviewers given their roles in the hospital medicine division, none had any type of reporting or supervisory relationship with them.

The interviews rapidly reached saturation, and the sentiments expressed in both focus groups were highly similar. No follow-up/repeat data collection was conducted. Recordings were transcribed; field notes were not taken during the interviews, and the transcripts were not provided to participants for content checking.

The same individuals who conducted the interviews and focus groups performed the qualitative data analysis using Dedoose software. We used a qualitative content analysis approach [11] using the interview/focus group guide as a starting point for deductive coding (analyzing data by question), followed by inductive analyses of specific concerns that were raised repeatedly in the interviews and focus groups. We used these themes to organize content data analysis and assigned them codes (see Table 3 for codes and definitions). These included perceived sufficiency of existing handoff mechanisms (chart notes and Epic chat), particularly within the specific hospital context; the importance of handoffs vis-à-vis patient safety; specific positive and negative aspects of both the I-PASS tool and the use of Epic chat/chart notes; frustration at time-consuming documentation in cases where it had little perceived utility; and concerns about “buy-in” by other providers, particularly night cover providers. The transcripts were coded inductively with codebook revision until interrater agreement was high (κ = 0.82).

**Table 3:** Qualitative analysis codes and definitions.

Title	Definition
Aspects-Poor	Aspects of a poor handoff process
Epic chat is sufficient	Discussion of perceptions that Epic chat is sufficiently effective for handoffs without additional components
Hospital setting	Discussions of the impact of the large, high-volume urban hospital setting and context for providers and patients. Includes discussions of other handoff systems
Needed	Perception that effective handoffs are necessary for patient safety
Night cover	Perceived use and acceptance of handoffs processes by night cover staff
Notes are enough	Discussion of perceptions that chart notes are sufficiently effective for handoffs without additional components
Quality improvement project raised awareness	Observations of how the I-PASS-based pilot project raised awareness of the need for handoffs
Suggestions for improvement	Suggestions for improvement or descriptions of an ideal system

Ethics approval and consent to participate

The protocol for this study received approval from Emory University Institutional Review Board (approval number: 00003615). All participants provided informed consent before interviews and focus groups.

## Results

Perceived necessity of handoff procedures for patient safety

Participants noted repeatedly that handoff processes are necessary to ensure patient safety, especially in the context of a high-volume institution with many cross-cover providers, and described several benefits of efficient handoffs, including heightened awareness of specific patient needs (see Table 4 for illustrative quotes). Despite concerns of feasibility of implementation in the form of the I-PASS-based tool, many agreed that if it is necessary for patient safety and helpful to both day and night providers, they are willing to put in the extra time to complete handoff procedures.

**Table 4:** Illustrative quotes for the “perceived necessity of handoff procedures for patient safety” thematic code.

Theme(s) expressed by participants	Sample quotes
Handoffs are necessary for patient safety	“So, I would [complete a handoffs process] for anticipatory guidance and for watchers [i.e., an individual who is at elevated risk of clinical deterioration] and follow up; those would be the three things. And I think that helps with patient safety and I think it helps with the night team workflow.”
	“I would buy into something if it’s better for patient safety. If they told me this specific tool is very helpful, then even if it meant more work for me, I would make sure to do that.”

The QI project raised awareness and changed behavior

Many attributed greater awareness of standardizing handoffs to our QI project; the project raised hospitalists’ awareness of the need for intentional handoffs processes, and helped them think through and categorize information to share with cross-cover providers (see Table 5 for illustrative quotes). Although once they were mindful of a handoff process, Epic chat was generally what they used. Several participants noted that handoff processes would need to be mandatory and monitored to ensure effective use.

**Table 5:** Illustrative quotes for the “quality improvement project raised awareness and changed behavior” thematic code.

Theme(s) expressed by participants	Sample quotes
Project raised awareness of handoffs	“I like that it at least got people thinking about handoffs, you know? … So even if they weren’t using the handoff tool, they were thinking about handoffs, and they were sending handoffs through the Epic chat when they weren’t before.”
	“It made you just very, very hyper cognizant of each of your patients, so you actively sat down and had that thought process of, ‘What do I need to tell the night provider about my patient? Do I need to…’ Well, I guess you had to say something [in the period before implementation of the tool], but it [the handoffs tool] really did help with your thought process actively, regarding what exactly you wanted to say.”
Any process would need to be mandated to change behavior effectively	“People have to stop asking doctors like, ‘Hey, can you please do this?’… Have someone follow up and say, ‘Hey, you have not done this. You need to do this. This is an expectation.’”

Feasibility of a written tool in the large, high-volume urban context

The large, high-volume urban hospital context with many cross-covers and moonlighting providers was repeatedly cited as a barrier by participants (see Table 6 for illustrative quotes). While participants saw the need for some sort of cross-communication between the day and night teams, the high volume of patients presented a significant barrier. Participants mentioned other systems they used before/elsewhere (e.g., verbal handoffs for each patient during residency), but few felt that systems that required documentation for every patient would be feasible. The piloted tool was perceived to necessitate extra steps, consolidation of information from multiple sources, and written sign-out for all patients regardless of condition, and was thus considered by some to be impractical and unlikely to be used. Participants described several aspects of various handoff processes, namely, the I-PASS tool, Epic chat, verbal signoffs, that were frustrating or ineffective. Lack of a standardized process was noted repeatedly as a barrier to utility.

**Table 6:** Illustrative quotes for the “feasibility of a written tool in the large, high-volume urban context" thematic code.

Theme(s) expressed by participants	Sample quotes
High patient volume as a barrier	“We had to do the whole handoff, the template, the tool that we had to use, we had to put that for every patient. And then we had to separately also sign out very sick patients to oncoming cross covers, because we have multiple cross covers and that change every three to four hours.”
Tools that require extra steps are unlikely to be adopted by users	“There’s such a volume of the patients that there needs to be a way to definitively track it for who’s sick, who's not sick … what we’re basically trying to come up with is what is the systematic ways to keep track of everyone so that stuff doesn’t go down overnight and people are best attended to.”
	“For the overnight people, there’s just so many, and [a handoffs tool should make it easier] for them to be able to kind of triage…When they’re covering for 100 patients or something, [they need to know] that the day provider was worried about this person, ‘let’s go take a look,’ versus not.”
	“We have to find out a balance where we can sign out the very sick patients, acute patients who need care, so it’s not too overwhelming for the provider who’s coming in cross covering as well. Because I’ve seen in past when I’ve tried to sign on almost all the patients, it gets really overwhelming and that’s too much information.”
Standardized processes are needed	“Handoffs are very user dependent, correct? Like my way of handing off is not same as the other person, or the information I’m looking for is probably/may not be there for the other person. Unless we standardize the whole process, it’s very difficult to expect anything out of that.”

Participant preferences for existing tools/systems

Most participants stated their belief that EMR notes/Epic interactive chat features (i.e., existing mechanisms for informal handoff communications) were sufficient in their current state, or could be made adequate with standardization and explicit requirements for use (see Table 7 for illustrative quotes). Several participants noted that they valued the “closed loop” aspect of the existing chat system. While some participants felt that Epic chat was an effective tool for handoffs, others noted that it was difficult to keep track of which group chat was specific to which patients, that the stream of messages could be overwhelming during time away from the job, and that there was no easy way to triage chat messages by priority. Several participants noted that, despite their belief that a handoff process was necessary, the I-PASS-based tool required too much time with too little perceived benefit. Participants noted that even if they initially utilized the tool during the pilot period, they frequently defaulted to existing systems (i.e., use of progress notes and Epic chat without manual input into the I-PASS-based tool).

**Table 7:** Illustrative quotes for the “participant preferences for existing tools/systems” thematic code.

Theme(s) expressed by participants	Sample quotes
Current systems are adequate	“I think what we have now in place is good. Using [chart notes supplemented with] the Epic chat to relay important information, I think is how I would do it. I think it can get very burdensome. Because at the end of the day, if there’s something active going on and you get paged about it, you end up opening the chart anyway.”
	“I think Epic chat message is ideal, straightforward, and for me, most importantly, you can connect it directly to the patient you’re talking about … [the I-PASS handoffs tool], that’s a longer stretch because you have to go out of the chart or go click on a few different things to find what’s been signed out to you.”
“Closed loop” advantage of the existing system	“I actually found it [the tool] challenging as well, as far as time management, and I often didn’t quite know exactly what I was supposed to do. So, I’d have my daily progress note, and then if I was really concerned about someone, I would still use Epic chat because I really like the closed loop communication.”
	“The Epic chat is pretty much how we as the hospitalist effectively hand off any patient who we’re truly worried about.”
Participants frequently defaulted to existing systems	“The information that you need does not jump out at you. I mean, I understand, ‘Here, you want to put patients are DNR, DNI,” [and] what the patient needs, but all of that information can also be found easily on Epic.”
	“I think I half-heartedly tried for a couple months and then very quickly gave up because I found that I was doing a ton of work with very little to no improvement in handoff.”

Importance of buy-in from providers, particularly night cover providers

Participants expressed that the utility of handoff processes for cross-cover and nighttime providers should be the highest priority, and that doing so would improve patient safety (see Table 8 for illustrative quotes). They shared their feelings of frustration when they took extra time to document information that they knew or suspected was not used by nighttime providers. Further, participants perceived that night cover staff who were unfamiliar with existing processes were unlikely to quickly implement any highly complex processes.

**Table 8:** Illustrative quotes for the “importance of buy-in from providers, particularly night cover providers” thematic code.

Theme(s) expressed by participants	Sample quotes
Frustration that time-intensive information entry was not utilized	“I think you need buy-in, not only from the nocturnist, but also the day shift people. I think that a lot of times people would do it on their first day of service and then they wouldn’t update it. I think it was viewed as just another thing to check off, that’s another form of documentation that we are being subjected to.”
	“I just didn’t think there was so much buy-in. I would write in the handoff, but that would require the night person to go then look at the handoff, as opposed to having a chat that already had them included. So, they would have to go search for my stuff in the handoff. And if they weren't bought into the system, I would find that the things that I would recommend were not done.”
Night cover providers need simple systems without need for many steps	“If I was working at night, I would never want to have to open a patient’s chart. I would just want to be able to look and to say, ‘Okay, these are the patients who are on team R’ and have a dropdown list.”

Suggestions for improving handoff processes

Participants had several suggestions for improvements (see Table 9 for an illustrative quote). They indicated that written handoffs should be limited to patients who are “watchers,” have anticipatory guidance, or have follow-up required, and should only include structured pertinent information. Many noted that EMR-integrated chat (Epic chat, in this case) allows for closed loop communication and should be used to alert the other provider, who can then review the patient chart and/or updated progress note as an already established standard operating procedure. Feedback from and collaboration with nighttime providers was viewed as paramount in implementing a successful handoff process. Providers shared that handoff tools should be streamlined onto one window in Epic (or other EMR), avoid multiple windows to click through, and have the entire list of patients and updates for each all in the same place. They recommended that handoff tools be integrated into the daily patient workflow in EMRs by automatically triggering a pop-up window with specific buttons to click to categorize the patient case and easy access to guidelines or things to look out for. They noted that, ideally, handoff tools can be helpful for anticipatory guidance in EMRs; for example, the blue “Internal Medicine” sticky note attached to the patient chart in Epic can also be used for focused handoffs or chronic anticipatory guidance.

**Table 9:** Illustrative quote for the “suggestions for improving handoff processes” thematic code.

Theme(s) expressed by participants	Sample quote
Suggestions for a successful handoff tool	“I think [what would help would be] buy-in, or a little bit more collaboration with a night crew for what is feasible before developing a tool and what they’re actually going to use as far as their night workflow. And they may have done that, and this may have been the best solution—[but] it just like, it didn’t feel that way to the group.”

## Discussion

Standardized approaches (e.g., the I-PASS) can improve patient safety [4-6], provider communication, and perceptions of quality care [7]. While reductions in medical errors and improved patient satisfaction after implementation of the I-PASS bundle have been reported in residency programs [2,8], we sought to qualitatively assess sustainability of components of the I-PASS tool with more experienced physicians in a large, urban, safety-net hospital setting.

Focus group and interview participants expressed a consistent perception that handoffs are important for patient safety and provider efficiency, reflecting consensus in the literature [1-3]. However, they also felt that handoff processes should minimize extraneous steps and avoid redundancy where possible. Shift handoffs should be standardized, including a formal expectation of which format will be used, which patients to hand off, what key information should be shared, and follow up. Disseminating successes and lessons learned may provide opportunities to share best practices that foster optimization of handoff processes.

There were limitations to our study. This was a single institution study. This pilot was implemented during the COVID-19 pandemic, and there were multiple new providers as well as providers who were moonlighting to cover additional service needs during the pilot period and soon afterwards that may have impacted the attitudes and perceptions of this project. Further, there were fewer nocturnists compared to daytime providers among study participants. This was, in part, due to the overall lower number of nocturnists in our hospitalist group and may have introduced bias in the collected perspectives that informed our results.

Finally, as there has been no standardized process in place at our institution following the pilot (though trainees use a standardized process, the direct care attending hospitalists who work with them do not), cross-cover providers rely primarily on the Epic chat feature and providers’ notes for information exchange, and view these as sufficiently effective that they are not incentivized to use another process. We do not at present have quantitative data with which to assess impacts on patient satisfaction or other metrics.

Strengths of this study include its implementation within a newer hospital medicine group, which provided an opportunity for process implementation and cultural change, including regularly scheduled group meetings for faculty development sessions. Additionally, there was strong collaboration with the informatics team to implement structural changes to the EMR to foster use of I-PASS sign-out format. While there has been previous research on use of I-PASS among attending physicians [12,13], these studies were conducted in a critical care unit rather than a hospital floor and focused on handovers at the end of rotations/service periods rather than individual shifts.

Hospitalist providers in our study found that the Epic chat function was the highest-yield shift handoff tool to convey information, but they were challenged by the constant real-time messaging. We found several Epic system-specific workarounds that can likely be adapted for analogous systems. To improve this function in a future iteration, Epic can be set to time-release where providers can add notes to their chat messages throughout their shifts, but the messages do not send to the other providers until the scheduled end of shift. Allowing users to name their Epic chats is an alternative option to improve organization and workflow. Yet another option is to pin the Epic chat messages to the patient charts or allow messages to be tagged as part of the handoff process. Educating providers on Epic chat functionality to minimize cell phone notifications is critical in achieving work-life balance.

The findings of our study may not be generalizable to all hospital medicine settings (e.g., smaller, more rural, or community contexts), as our facility is a large, direct care hospital in an urban setting, which entails a high volume of patients with a wide range of acuity. However, many of the conditions experienced by our providers are universal to hospital medicine practice and may therefore be informative for similar efforts to implement components of the I-PASS bundle. Any such implementation, whether of specific I-PASS bundle components or the entire bundle, will require leadership buy-in and effective education, and healthcare systems should employ ongoing process improvement and change management principles when implementing a uniform handoff process across multiple service lines.

Emphasizing the importance of educating providers that handoffs are a standard documentation expectation and consistently providing feedback on adherence may support successful implementation of similar projects. Future research should focus on effective forms of education and feedback, as well as developing a more seamless integration of handoff creation into the daily workflow of the provider.

## Conclusions

While the participants perceived the I-PASS-based tool to be suboptimal in several aspects, they felt that the handoff QI pilot raised overall awareness and use of handoff processes, which they felt was important for patient safety. Thus, implementation of the I-PASS bundle components may require contextual adaptation. In this study, participants recommended that feasible handoff processes provide necessary information quickly for cross-cover providers, but do not require similar details for stable patients. Most participants reported that existing EMR chat functions along with notes were adequate and more efficient. They also recommended standardizing format and processes; obtaining buy-in from all patient care providers; education on efficient use of tools; and formal, explicit expectations that providers complete handoffs.

## Appendices

Focus group and interview question guide

Introduction/Welcome statement

Shift handoffs are a way to relay patient information between primary and cross-cover providers.

1. Should there be a shift handoff process? a. Why or why not?

2. If you were to design a shift handoff process, what would it look like ideally?

3. What is your experience with shift handoffs prior to the implementation of this QI project?

4. What did you think of the shift handoff project implemented for the Grady hospitalist group? a. What worked well? What did not work well (would you change)?

5. We saw an improvement in satisfaction with shift handoffs during the duration of the project - why do you think this was?

6. We noticed a lower uptake of written handoff - what were the reasons you had to use this or not use this?
